# Domains or no domains? That is the question: insights from asymmetric bilayer models into plasma membrane lipid organization

**DOI:** 10.1007/s12551-026-01468-1

**Published:** 2026-09-30

**Authors:** Thais Azevedo Enoki

**Affiliations:** Universidade de São Paulo, Institute of Physics, Department of Experimental Physics, São Paulo, SP 05508-090 Brazil

**Keywords:** Plasma membrane, Model membrane, Phase diagrams, Coexistence of liquid disordered and liquid ordered phases, Asymmetric bilayers

## Abstract

The mammalian plasma membrane is characterized by its pronounced lipid asymmetry, including the exceptional enrichment of sphingomyelin (SM) in the exoplasmic leaflet. How this asymmetry influences cholesterol distribution and membrane organization remains a central and unresolved question in cell biology. The lipid raft hypothesis has long provided the prevailing framework, proposing that cholesterol and SM assemble into dynamic liquid-ordered nanodomains that compartmentalize proteins and regulate membrane trafficking, signaling, and host–pathogen interactions. More recently, a phospholipid (and cholesterol) imbalance model offers an alternative view, proposing that the exoplasmic leaflet contains substantially fewer phospholipids but is correspondingly enriched in cholesterol, potentially shifting its composition toward a uniformly liquid-ordered state. In this review, we examine these two conceptual frameworks in light of recent advances in membrane lipidomics and asymmetric model membranes. We discuss the experimental evidence supporting both models and emphasize that many observations attributed to a large phospholipid imbalance are not unique to that interpretation but can also be explained by membrane lateral lipid heterogeneity. We further highlight methodological considerations in enzymatic lipid digestion and cholesterol quantification, underscoring the need for additional experimental validation of leaflet-specific lipid composition. A central theme of this review is the role of interleaflet interactions. Experimental studies using asymmetric model membranes demonstrate that membrane domains in one leaflet can induce lateral organization in the opposing leaflet, even when the latter lacks the lipid composition required to form liquid-ordered domains independently. We suggest that interleaflet coupling provides a physical mechanism that reconciles lipid domain (lipid raft) formation with the compositional asymmetry of the plasma membrane. Rather than viewing the lipid raft and phospholipid imbalance models as mutually exclusive, we propose that their apparent differences depend critically on the magnitude of phospholipid and cholesterol asymmetry and on the strength of transbilayer coupling. Understanding how these factors collectively govern membrane organization will be essential for developing a unified physical description of the plasma membrane and for elucidating the mechanisms underlying cholesterol homeostasis, membrane protein regulation, and cellular signaling.

**Supplementary Information:**

The online version contains supplementary material available at https://doi.org/10.1007/s12551-026-01468-1.

## Lipid repertoire in biological membranes

Biological membranes consist of a diverse range of lipids, which can be categorized into several distinct groups: glycerolipids, sphingolipids, sterols, and glycolipids. These molecules encompass a diverse range of molecular species that vary in fatty acyl chain length, the presence of double bonds, and their chemical linkages. Mass spectrometry experiments have unveiled thousands of lipid species within a single cell (van Meer et al. 2008; Brügger 2014; Lorent et al. 2020; Qian et al. 2025). This diversity is conserved across different organisms, from mammals to yeast, indicating that a rich and complex lipid repertoire is a fundamental aspect of biological membranes.

We can briefly summarize lipid synthesis and the differences in lipid composition between cellular organelles and the cell plasma membrane.

## Membranes and their lipids

The endoplasmic reticulum (ER) is the leading organelle for producing most of the structural lipids in eukaryotic cells. It synthesizes phospholipids, including phosphatidylcholine, phosphatidylethanolamine, and phosphatidylserine. The ER also produces cholesterol (in mammals) and ergosterol (in yeast). Additionally, it produces triacylglycerols for energy storage, cholesteryl esters, ceramide (a precursor of sphingolipids), and dolichol, which is involved in protein glycosylation.

The Golgi apparatus produces and modifies lipids, particularly sphingolipids. It synthesizes several sphingolipids, such as sphingomyelin, glucosylceramide, lactosylceramide, and other complex glycosphingolipids, which are then transported to the plasma membrane. The Golgi also helps in making phosphatidylethanolamine from phosphatidylserine and assists in the final steps of phosphatidylcholine synthesis, which occurs partly in the ER and Golgi. Additionally, it sorts and distributes these lipids to different parts of the cell.

Mitochondria contains phosphatidylglycerol and cardiolipin, a unique lipid crucial for oxidative phosphorylation. Mitochondria also produce phosphatidylethanolamine, which is involved in the synthesis of lysophosphatidic acid, a component used in fat storage. It can synthesize and metabolize fatty acids for energy, import cholesterol from the endoplasmic reticulum (ER), and produce dolichol, which, as mentioned above, aids in protein glycosylation.

Early endosomes, which are responsible for endocytosis, have a composition similar to that of plasma membranes but change during maturation into late endosomes. Specifically, there is a decrease in sterols and phosphatidylserine and a significant increase in bis(monoacylglycerol)phosphate (BMP), which aids in multivesicular body formation, fusion, and sphingolipid breakdown. Specific phosphoinositides mark endosomal membranes: PI(4,5)P2 on the plasma membrane, PI3P on early endosomes, PI(3,5)P2 on late endosomes, and PI4P on the trans-Golgi network. These lipids help identify membrane compartments and recruit proteins involved in vesicle trafficking and cellular regulation. Despite their low quantity, PIPs play a crucial role in cell function (Han et al. 2020).

In this review, we focus on the plasma membrane of mammalian cells. Although significant progress has been made in lipidomics and membrane analysis, accurately defining the lipid composition of individual cellular membranes remains a major experimental challenge. Determining the lipid repertoire of the plasma membrane is particularly difficult because most analytical approaches require membrane isolation or enzymatic treatment, procedures that can compromise bilayer integrity, disrupt transbilayer lipid asymmetry, and ultimately lead to mixing of the lipid compositions of the two leaflets (Feigenson 2025). Moreover, different lipid extraction protocols and analytical methods often yield substantially different lipid profiles, highlighting the methodological variability that continues to complicate comparisons across studies.

A more reliable strategy is to examine the plasma membrane of red blood cells, which lack intracellular organelles and therefore eliminate the possibility of contamination from internal membrane systems (Verkleij et al. 1973; Lorent et al. 2020). Even in this simplified model, however, preserving the native transbilayer asymmetry during sample preparation is essential. Experimental protocols must be carefully designed to avoid perturbing lipid organization, as even subtle membrane damage can alter the apparent lipid distribution. These considerations are discussed in detail below.

A striking feature of the plasma membrane is its exceptionally high sphingomyelin (SM) content compared with other intracellular membranes (see Figure S1), which is by far the highest concentration of sphingomyelin (SM) in the cell. Apart from the Golgi apparatus, no other organelle is significantly enriched in SM, underscoring the importance of this lipid in conferring the unique structural and biophysical properties of the plasma membrane (van Meer et al. 2008). This enrichment is particularly important because the SM species predominantly found in the outer leaflet possess high melting temperatures (Tm), with gel-to-fluid phase transition temperatures typically exceeding 40 °C in pure bilayers. As a result, these lipids remain highly ordered under physiological conditions and strongly influence the membrane's structure and consequently its physical properties (Feigenson 2006).

An equally important characteristic of the plasma membrane is its high cholesterol content, which typically accounts for 30–40 mol% of total lipids. Cholesterol is indispensable for numerous cellular processes, including membrane organization, signaling, and mechanical stability. Unlike phospholipids, cholesterol contains only a small hydroxyl group (-OH) as its polar head and therefore cannot self-assemble into a stable bilayer. Instead, its incorporation into membranes depends on neighboring phospholipids, whose head groups shield cholesterol from the aqueous environment while accommodating its hydrophobic sterol ring within the membrane core (Huang et al. 1999).

In particular, cholesterol exhibits a strong affinity for sphingomyelin (SM) and other high-melting-temperature (high-*T*m) lipids, with which it can form tightly packed yet laterally mobile assemblies. These interactions favor the formation of the liquid-ordered (Lo) phase, a distinctive membrane state that combines a high degree of molecular order with substantial lateral mobility, thereby distinguishing it from both the more rigid gel phase and the less ordered liquid-disordered (Ld) phase.

Importantly, SM itself is distributed highly asymmetrically across the plasma membrane, being strongly enriched in the outer leaflet. This remarkable and evolutionarily conserved asymmetry suggests an important role for SM in plasma-membrane organization. As discussed throughout this review, experimental and theoretical evidence supports a close relationship between SM and the high cholesterol content characteristic of the plasma membrane. SM may promote the formation of SM–cholesterol-enriched ordered domains and/or provide a favorable molecular environment for accommodating cholesterol within the membrane, thereby reducing unfavorable cholesterol self-association while maintaining membrane integrity. These mechanisms are not mutually exclusive and provide a useful framework for understanding the functional significance of the strong enrichment of SM in the outer leaflet and its association with cholesterol.

When translating these concepts to model membranes, however, it is important to recognize that biological membranes are considerably more compositionally complex than the simplified lipid mixtures typically employed in experimental systems. Lipidomic analyses have shown that mammalian plasma membranes are generally enriched in SM and cholesterol and display lower overall lipid unsaturation than crude cellular membrane fractions, which comprise membranes originating from multiple subcellular compartments rather than the plasma membrane alone, (Symons et al. 2021). Nevertheless, biological SM does not correspond to a single molecular species but rather comprises a heterogeneous population differing in both *N*-acyl-chain length and degree of unsaturation. Furthermore, the abundance and molecular-species distribution of SM vary considerably among different cell types (Symons et al. 2021).

The degree of unsaturation reported by lipidomic analyses also requires particular care when interpreting SM molecular species. Most naturally occurring SM contains a sphingosine backbone with a **trans** double bond, which perturbs hydrocarbon-chain packing much less strongly than the **cis** double bonds commonly found in unsaturated glycerophospholipid acyl chains. Consequently, the presence of one or more double bonds in an SM molecular species does not necessarily imply a low melting temperature or strongly disordered lipid packing. Brain sphingomyelin provides an illustrative example: although it is a heterogeneous mixture containing molecular species with different degrees of unsaturation, it retains a relatively high and broad chain-melting transition. This behavior reflects not only double-bond geometry but also the contribution of long and very-long *N*-acyl chains and the strong intermolecular interactions characteristic of SM, including hydrogen bonding. Thus, the nominal degree of unsaturation alone is insufficient to predict the ordering propensity or phase behavior of SM-rich membranes.

For this reason, the simplified compositions commonly employed in model-membrane studies should not be regarded as exact replicas of the plasma membrane. Rather, they provide experimentally controlled systems in which specific physical principles—such as SM–cholesterol interactions, phase behavior, and interleaflet coupling—can be isolated and systematically investigated. The insights obtained from these simplified systems can then provide a foundation for progressively incorporating the greater compositional complexity characteristic of biological membranes.

Before examining the models describing the lipid composition on the outer leaflet of the plasma membrane, it is essential to consider another defining characteristic of the plasma membrane: its transbilayer lipid asymmetry (Verkleij et al. 1973; Lorent et al. 2020). This fundamental feature has profound implications for membrane organization and must be taken into account when evaluating any model of the membrane and its interactions.

## Plasma membrane asymmetry

Cells continuously expend metabolic energy to preserve membrane asymmetry, underscoring its physiological importance. For example, P4-ATPases (flippases) are ATP-dependent lipid transporters that selectively translocate phospholipids from the exoplasmic to the cytoplasmic leaflet, thereby establishing and maintaining the characteristic lipid composition of each monolayer. Extensive genetic, biochemical, and physiological studies have demonstrated that P4-ATPases, together with their indispensable β-subunit CDC50, are required for membrane lipid asymmetry across diverse organisms. Disruption of these transport systems compromises membrane organization, impairs cell growth and development, and has been implicated in numerous human diseases (Li et al. 2024). Furthermore, the collapse of plasma membrane asymmetry is a well-established hallmark of apoptosis, where the exposure of phosphatidylserine on the cell surface serves as a signal for the recognition and clearance of dying cells.

If cells invest considerable metabolic energy to maintain transbilayer lipid asymmetry, it follows that the asymmetric localization of individual lipid species must also have important functional consequences. This reasoning is particularly compelling for sphingomyelin (SM), which is concentrated almost exclusively in the outer leaflet of the plasma membrane. Such a highly conserved and energetically maintained distribution is unlikely to be incidental. Instead, it strongly suggests that SM fulfills a specialized role in determining the structural and functional properties of the plasma membrane. The fundamental question, therefore, is not simply why SM is abundant, but why evolution has selected its enrichment specifically in the exoplasmic leaflet.

**Fig. 1:**
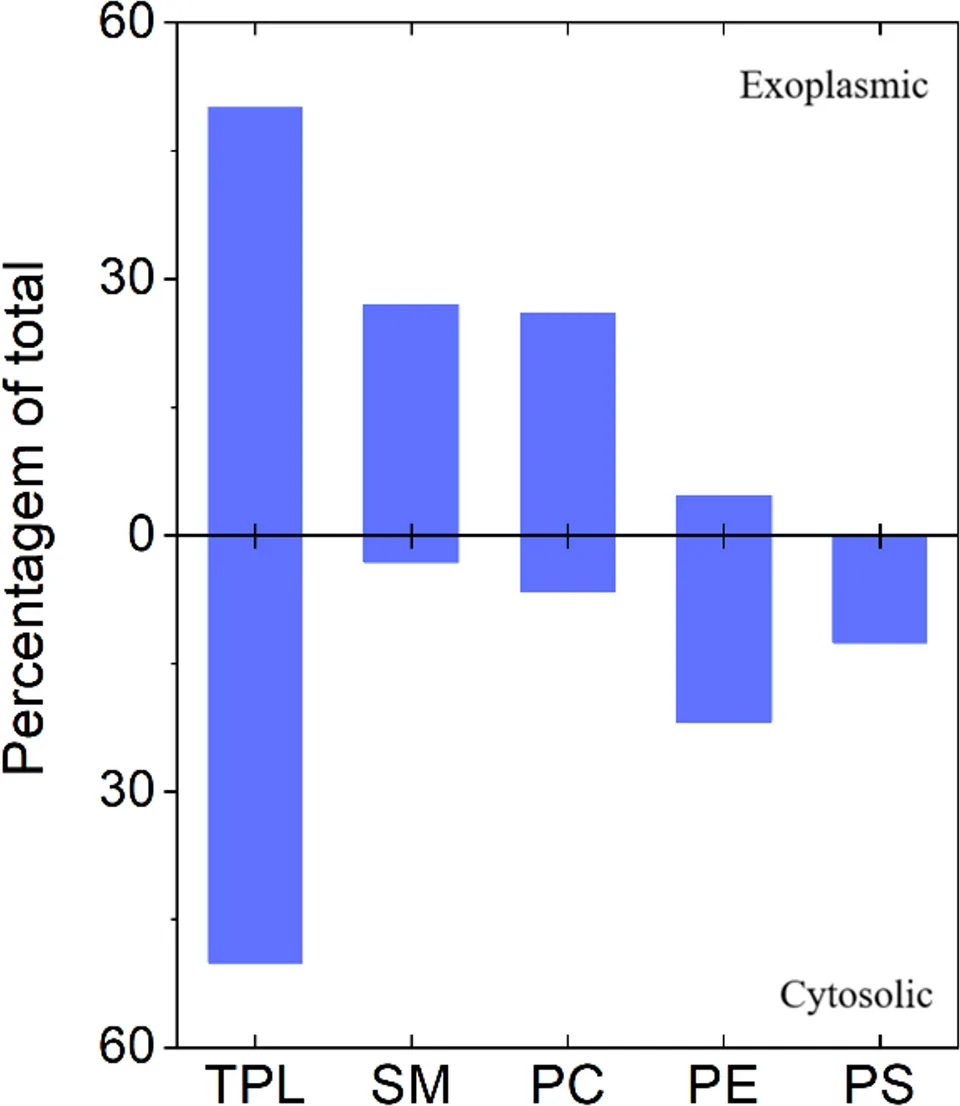
The asymmetry of the plasma membrane of mammalian cells. The graph represents the percentage of lipids in the exoplasmic (outer) leaflet and cytosolic (inner) leaflet of the plasma membrane. The horizontal axis represents sphingomyelin (SM), phosphatidylcholine (PC), phosphatidylethanolamine (PE), and phosphatidylserine (PS). TPL means total phospholipids—data sourced from (Verkleij et al. 1973)

Figure 1 summarizes the current understanding of plasma membrane lipid asymmetry, building on the pioneering work of (Verkleij et al. 1973), which established the asymmetric distribution of phospholipids across the plasma membrane. More recently, the comprehensive lipidomic analyses of (Lorent et al. 2020) have substantially refined this picture by quantifying the lipid composition of each leaflet and revealing the remarkable diversity of acyl chain species that contribute to membrane asymmetry.

One of the most striking observations is that the cytoplasmic (inner) leaflet contains polyunsaturated phospholipids, where the number of double bonds on the acyl chains is approximately twice that observed in the exoplasmic (outer) leaflet (Lorent et al. 2020). This enrichment in unsaturated acyl chains reduces lipid packing, resulting in a more fluid and dynamic membrane environment. Such increased fluidity is likely to facilitate a range of essential cellular processes, including the lateral diffusion of membrane proteins, the rapid reorganization of signaling complexes, membrane remodeling, and vesicular trafficking.

Determining the transbilayer distribution of cholesterol remains a major experimental challenge, and no consensus has yet emerged regarding its partitioning between the two leaflets of the plasma membrane (Gibson Wood et al. 2011; Varma and Deserno 2022; Zhu et al. 2024).

The asymmetry in the distribution of sphingomyelin and polyunsaturated phospholipids also suggests that the two leaflets possess fundamentally different biophysical properties and could therefore exhibit distinct phase behaviors. The exoplasmic leaflet, enriched in sphingomyelin and cholesterol, provides an ideal environment for the formation of liquid-ordered domains (in a coexistence-of-phases context) or a liquid-ordered phase. In contrast, the cytoplasmic leaflet is dominated by polyunsaturated phospholipids, whose reduced packing density favors a more fluid membrane with enhanced lateral diffusion. Consequently, the plasma membrane should not be regarded as a bilayer of two similar leaflets but rather as two chemically and biophysically distinct monolayers that are somewhat coupled while maintaining markedly different lipid compositions and dynamic properties.

These marked differences between the two leaflets raise a fundamental question: if each leaflet possesses a distinct lipid composition and, consequently, different phase behavior, how are they mechanically and functionally coupled? To what extent does the organization of one leaflet influence that of the other? Can the plasma membrane be understood in terms of interleaflet coupling, whereby structural or dynamic changes in one monolayer propagate across the bilayer?

These questions are central to our understanding of plasma membrane organization. Any comprehensive model of membrane structure—whether based on lipid rafts or alternative frameworks—must account not only for the properties of the individual leaflets but also for the mechanisms by which they communicate and influence one another. Interleaflet coupling is therefore not a secondary consideration but a fundamental constraint that any successful model of plasma membrane organization must explain.

Thus, in this review, we examine and contrast two distinct models for plasma membrane organization. We begin with the widely accepted lipid raft hypothesis and then introduce an alternative framework, which we refer to as the phospholipid (and cholesterol) imbalance model (Doktorova et al. 2025).

## Lipid rafts hypothesis

The modern concept of lipid rafts originated from pioneering studies of epithelial cells, which sought to explain how distinct lipid compositions are established and maintained in the apical and basolateral plasma membranes. Using MDCK cells as a model system, these studies demonstrated that lipid sorting begins in the Golgi apparatus, where specific lipid species are selectively packaged into transport carriers. In particular, sphingolipids are preferentially incorporated into the luminal (future exoplasmic) leaflet of the Golgi membrane and subsequently delivered to the apical plasma membrane (Simons and van Meer 1988). This selective trafficking provides compelling evidence that membrane lipid asymmetry is actively generated during membrane biogenesis. Consequently, the Golgi apparatus functions as a central organizer of membrane lipid composition, ensuring that epithelial cells maintain the specialized membrane architecture required for their physiological functions.

These observations laid the conceptual foundation for the lipid raft hypothesis. As the model evolved, lipid rafts came to be defined as small membrane domains enriched in sphingolipids and cholesterol that exist in a liquid-ordered (Lo) state embedded within the more fluid liquid-disordered (Ld) membrane (Simons and Ikonen 1997; Rietveld and Simons 1998; Simons and Ehehalt 2002; Rajendran and Simons 2005; Lingwood et al. 2008; Kaiser et al. 2009). Unlike gel phases, the liquid-ordered phase combines high molecular packing with lateral mobility, creating membrane environments with distinct physical properties. These domains were proposed to selectively recruit or exclude membrane proteins, thereby regulating protein localization, intermolecular interactions, and signaling. The lipid raft hypothesis rapidly gained acceptance because it provided a unifying framework to explain a broad range of cellular processes, including receptor activation, membrane trafficking, signal transduction, and membrane remodeling.

Despite its broad biological relevance, direct visualization of membrane rafts in living cells has remained a major experimental challenge owing to their small size (typically < 200 nm), transient lifetime (milliseconds), and highly dynamic behavior. As highlighted in recent reviews by (Kusumi et al. 2020) and (Nieto-Garai et al. 2022), much of the historical controversy surrounding lipid rafts stems from the technical limitations of available experimental approaches rather than from a lack of evidence for membrane heterogeneity. These authors propose that membrane rafts should be regarded as transient, liquid-like molecular assemblies arising from cooperative lipid–lipid and lipid–protein interactions, emphasizing that their characterization requires multiple complementary biophysical and biochemical techniques rather than any single experimental method.

Recent advances in super-resolution microscopy have begun to overcome these limitations and provide increasingly direct support for this view. Using STED nanoscopy, (Eggeling et al. 2009) demonstrated that sphingomyelin, GM1, and GPI-anchored proteins undergo transient, cholesterol-dependent confinement within membrane regions smaller than 20 nm, whereas phosphoglycerolipids diffuse freely. Complementing these observations, (Lingwood et al. 2008) showed that the plasma membrane is intrinsically poised to undergo raft-phase coalescence, with nanoscale heterogeneities rapidly assembling into larger cholesterol-dependent functional domains following GM1 clustering. Together, these studies indicate that membrane rafts are highly dynamic nanoscale assemblies that can reorganize in response to physiological stimuli, providing a versatile platform for membrane compartmentalization and cell signaling.

Moreover, the lipid raft hypothesis has gained considerable support from experiments using well-defined model membranes, in which lipid composition and experimental conditions can be precisely controlled (Feigenson 2006, 2008; van Meer et al. 2008). Studies of simplified ternary mixtures consistently demonstrated the coexistence of two liquid phases: a cholesterol- and sphingolipid-rich liquid-ordered (Lo) phase and a liquid-disordered (Ld) phase enriched in unsaturated or low-melting-temperature (low-Tm) phospholipids (Petruzielo et al. 2013). These experiments provided direct evidence that lateral phase separation can occur under controlled conditions and established a robust biophysical foundation for the lipid raft hypothesis.

**Fig. 2:**
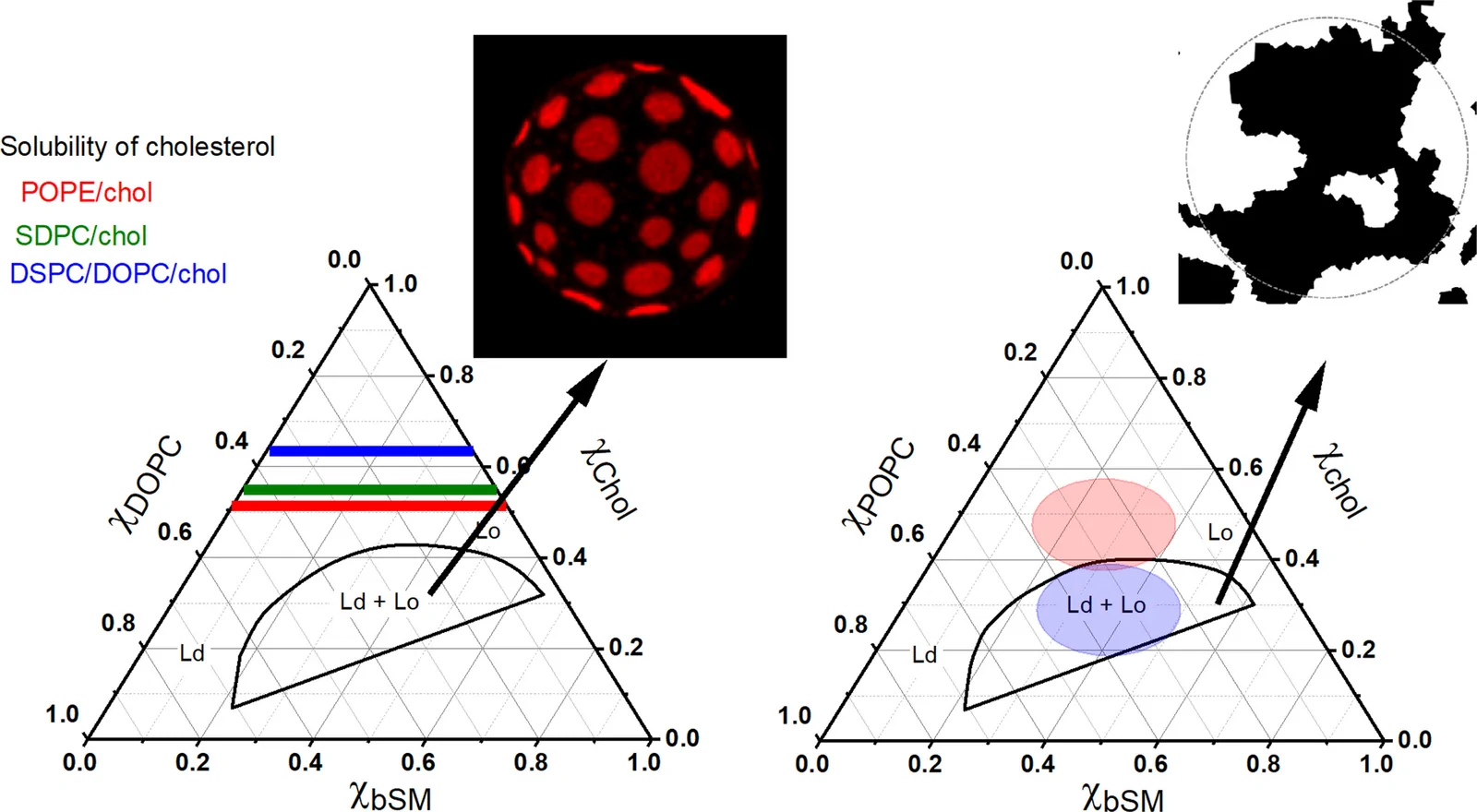
Phase diagrams of representative ternary lipid mixtures illustrating membrane phase behavior in model systems. **(a)** Phase diagram of the brain SM/DOPC/cholesterol mixture. The horizontal lines indicate the reported cholesterol solubility limits for different lipid systems: DSPC/DOPC/cholesterol (blue), SDPC/cholesterol (dark green), and POPE/cholesterol (red). Within the Ld/Lo coexistence region, phase separation gives rise to micron-sized domains that can be directly visualized by fluorescence microscopy, as illustrated in the micrograph (scale bar, 5 μm). **(b)** Phase diagram of the brain SM/POPC/cholesterol mixture. The shaded regions indicate lipid compositions representative of the exoplasmic leaflet of the plasma membrane: the blue region corresponds to membranes containing approximately 30–40 mol% cholesterol, whereas the red region represents the higher cholesterol content proposed by the phospholipid imbalance model. In this system, phase separation occurs on the nanometer scale and is therefore below the resolution limit of conventional optical microscopy. The inset shows representative molecular dynamics simulations of a DPPC/DUPC/PUPC/chol membrane, illustrating the nanoscale organization of Ld and Lo nanodomains (Usery et al. 2017)

To ensure clarity and facilitate a comprehensive understanding, it is important to define the terminology used throughout this review. The term “lipid raft” has been used in a variety of contexts to describe membrane heterogeneity in living cells, and there is currently no consensus regarding its precise size, lifetime, or lipid composition. Consequently, the term has occasionally been applied to distinct, and not always equivalent, membrane phenomena. On the other hand, studies using model membranes provide a well-defined thermodynamic framework in which liquid-ordered (Lo) and liquid-disordered (Ld) phase coexistence can be rigorously characterized under controlled conditions, as the regions described in the phase diagram of Fig. 2. These experiments offer independent biophysical evidence that cholesterol- and sphingomyelin-rich domains are energetically favorable and may represent the physical basis of the membrane heterogeneity proposed to exist in cells.

Accordingly, in this review we distinguish between thermodynamic phases and membrane domains. When discussing phase diagrams, we refer to the equilibrium coexistence of liquid phases in model membranes. In contrast, when referring to *domains*, we use the term in a broader sense to describe membrane regions whose composition and physical properties differ from those of their surroundings, regardless of whether they represent equilibrium phases or dynamic, transient structures. This distinction is important because membrane domains in cells may be continuously assembled, remodeled, and regulated while still sharing fundamental biophysical principles with the liquid-ordered domains observed in simplified model membranes.

In addition, a major strength of these model systems is that, despite their apparent simplicity, they capture the essential physicochemical features of the plasma membrane. Rather than attempting to reproduce the hundreds of distinct lipid species found in mammalian cells, the membrane lipidome is represented by a small number of functional classes: high-melting-temperature (high-Tm) lipids, low-Tm lipids, and cholesterol. This coarse-grained representation preserves the key molecular interactions that govern membrane organization, particularly the preferential association of cholesterol with high-Tm lipids, while greatly simplifying experimental interpretation. Remarkably, these minimal systems reproduce the membrane organization predicted for biological membranes, demonstrating that the emergence of liquid-ordered domains is a fundamental consequence of lipid interactions (Feigenson and Buboltz 2001; Veatch and Keller 2003; Chiang et al. 2005; Feigenson 2007; Mills et al. 2008; Heberle et al. 2010; Konyakhina et al. 2013; Petruzielo et al. 2013; Rayermann et al. 2017).

Figure 2 illustrates representative phase diagrams for two ternary lipid mixtures: brain sphingomyelin (SM)/DOPC/cholesterol (A) and brain SM/POPC/cholesterol (B) (Petruzielo et al. 2013). The principal difference between these systems lies in the low-melting-temperature (low-Tm) phospholipid. DOPC contains two unsaturated acyl chains, whereas POPC is a hybrid lipid containing one saturated and one unsaturated chain. Despite this seemingly modest structural difference, the two mixtures exhibit strikingly different lateral organizations. The DOPC-containing membrane forms coexisting liquid domains with characteristic sizes on the order of micrometers, whereas replacing DOPC with POPC reduces the characteristic domain size to the nanometer scale.

These observations illustrate the remarkable sensitivity of membrane organization to subtle changes in lipid molecular structure. Domain size emerges naturally from the balance of lipid-lipid interactions and the energetic cost associated with the interface between coexisting Ld and Lo phases. In particular, the line tension at the domain boundary, which is influenced by factors such as differences in lipid packing and hydrophobic thickness between the two phases, plays a major role in determining the equilibrium domain size (Baumgart et al. 2003; Usery et al. 2017).

Importantly, numerous experimental studies have shown that nanoscopic domains exhibit biophysical characteristics closely resembling those of their micron-sized counterparts, despite being below the resolution limit of conventional optical microscopy. Consequently, micron-sized domains observed in model membranes are widely regarded as experimentally accessible analogs of the nanoscale heterogeneity proposed to exist in biological membranes (Enoki et al. 2018; Feigenson and Enoki 2023).

Furthermore, estimates based on plasma membrane lipidomic data suggest that the overall lipid composition of mammalian plasma membranes lies within, or close to, the Ld/Lo coexistence region (Fig. 2 blue shaded region) identified in phase diagrams obtained by multiple independent research groups. Although such comparisons should be interpreted with appropriate caution given the complexity of cellular membranes, they suggest that controlled conditions required for liquid-phase coexistence are compatible with the native lipid composition of the plasma membrane. As a result, phase diagrams derived from simplified lipid mixtures continue to provide a valuable reference for interpreting membrane organization in living cells.

In addition, a notable biological justification for the lipid raft model is the strong association found between cholesterol-rich membrane regions and viral infections (Nguyen and Hildreth 2000; Liao et al. 2001). Numerous studies have demonstrated that HIV-1, influenza virus, and several other enveloped viruses preferentially associate with cholesterol- and sphingolipid-rich regions of the plasma membrane during critical stages of their life cycles (Dick et al. 2012). In HIV-1 infection, for example, viral assembly, budding, and release occur at cholesterol-rich regions of the host membrane, suggesting that these membrane environments provide favorable conditions for concentrating viral proteins and promoting efficient particle formation (Ono and Freed 2005).

Additional experimental evidence arises from cholesterol depletion experiments. Treatment of cells with methyl-β-cyclodextrin or 2-hydroxypropyl-β-cyclodextrin, which selectively extracts cholesterol from the plasma membrane, consistently results in a marked reduction in HIV-1 infectivity (Liao et al. 2001). Cholesterol depletion impairs viral attachment, decreases membrane fusion, inhibits syncytium formation, and strongly suppresses the production of infectious viral particles. Similar cholesterol dependence has been reported for numerous enveloped viruses, reinforcing the conclusion that membrane cholesterol is essential for efficient viral replication. Because cholesterol extraction also disrupts the formation and stability of liquid-ordered domains, these findings have frequently been interpreted as functional evidence supporting the existence of lipid rafts in living cells.

The lipid raft model has therefore become one of the dominant frameworks for explaining viral assembly. According to this view, viral structural proteins accumulate within cholesterol-rich membrane domains, where their local concentration promotes multimerization and virion assembly before budding from the plasma membrane. The ability of rafts to compartmentalize both lipids and proteins offers a compelling mechanistic explanation for how viruses efficiently organize their assembly machinery while exploiting the host membrane.

Despite its explanatory power, however, an important conceptual challenge emerges when plasma membrane asymmetry is considered. Classical lipid rafts are thought to arise from the strong interactions between cholesterol and sphingomyelin, the latter being almost exclusively confined to the exoplasmic leaflet of the plasma membrane. In contrast, the cytoplasmic leaflet contains essentially no sphingomyelin and is instead enriched in phosphatidylethanolamine, phosphatidylserine, and phosphatidylinositol species. If sphingomyelin is a principal driver of liquid-ordered domain formation, it becomes difficult to reconcile the lipid raft model with observations indicating that viral structural proteins assemble on the cytoplasmic leaflet of the plasma membrane. To address this apparent contradiction, several authors have proposed that cholesterol-rich domains distinct from classical sphingomyelin-containing rafts may also exist within the inner leaflet (Ono and Freed 2005; Favard et al. 2019). Such domains could provide the membrane organization required for viral assembly while differing fundamentally in composition and physical properties from the canonical rafts described for the outer leaflet.

It is important to recognize that the precise lipid composition and structural organization of lipid rafts in living cells remain incompletely defined. Lipid rafts were originally proposed to be membrane domains enriched in sphingomyelin (SM) and cholesterol. However, the pronounced transbilayer asymmetry of the plasma membrane raises important questions regarding its structure (Sezgin et al. 2017; Enoki and Feigenson 2019). Because SM is localized almost exclusively in the exoplasmic leaflet, do domains exist only on the outer leaflet? If so, how should the organization of the cytoplasmic leaflet be described, given that it lacks significant amounts of high-Tm lipids that are thought to stabilize the liquid-ordered phase?

In particular, an important unresolved question is how domain formation in the sphingomyelin-rich outer leaflet is coupled to the chemically distinct inner leaflet, and whether the organization of one leaflet influences the lateral organization of the other. Resolving this issue is essential to developing a comprehensive understanding of plasma membrane architecture, as any successful model must account not only for lateral membrane heterogeneity but also for the bilayer's asymmetry.

At the same time, the existence of membrane asymmetry should not be viewed as evidence against the lipid raft hypothesis. On the contrary, growing experimental evidence from asymmetric model membranes demonstrates that domain formation in one leaflet can induce corresponding lateral organization in the opposing leaflet, even when that leaflet lacks the high-melting-temperature (high-Tm) lipids traditionally associated with liquid-ordered phase formation (Lin and London 2015; Wang and London 2018; Enoki and Feigenson 2019, 2022; Feigenson et al. 2023). These findings suggest that interleaflet coupling can propagate membrane organization across the bilayer, giving rise to transbilayer domains in which the two leaflets differ in lipid composition and in the mechanisms underlying their organization, yet remain physically and functionally coupled.

As discussed in the following sections, this emerging framework provides a mechanism by which lipid domains can be reconciled with the intrinsic asymmetry of the plasma membrane, offering a more complete picture of membrane organization.

In the following section, we examine a recently proposed view of plasma membrane organization based on a phospholipid (and cholesterol) imbalance between the two leaflets (Doktorova et al. 2025). This model introduces an important perspective on membrane asymmetry and predicts that the exoplasmic leaflet is more densely packed and ordered. In contrast, the cytoplasmic leaflet remains comparatively fluid owing to its distinct lipid composition. Whether these observations are compatible with the existence of sphingomyelin- and cholesterol-rich domains depends, to a large extent, on the magnitude of the proposed cholesterol enrichment in the outer leaflet and its consequences for membrane phase behavior (see discussion below).

We favor a framework in which lipid domains are present and interleaflet coupling allows their organization to propagate across the bilayer, thereby influencing the chemically distinct cytoplasmic leaflet. Importantly, however, this interpretation does not necessarily conflict with the phospholipid imbalance model. The extent to which the two frameworks differ depends critically on the degree of phospholipid—and consequently cholesterol—imbalance between the leaflets. Therefore, before assessing the implications of this new model, we first discuss several observations whose interpretation does not necessarily require a large cholesterol imbalance and remains compatible with the existence of laterally heterogeneous domains.

## Phospholipid (and cholesterol) imbalance

A recent study has proposed a substantial phospholipid imbalance between the two leaflets of the plasma membrane. According to the authors, the cytoplasmic (inner) leaflet contains approximately 1.5- to 2.3-fold more phospholipids than the exoplasmic (outer) leaflet. To account for this asymmetry, the authors further propose that cholesterol is preferentially enriched in the phospholipid-deficient outer leaflet, effectively compensating for the reduced phospholipid density (Doktorova et al. 2025). Together, these observations form the basis of what we refer to as the phospholipid imbalance (and cholesterol imbalance) model.

An important implication of this model concerns the predicted phase behavior of the outer leaflet. When the proposed phospholipid imbalance exceeds approximately twofold, the resulting outer leaflet composition no longer falls within the liquid-ordered/liquid-disordered (Lo/Ld) coexistence region of representative model membrane phase diagrams (Fig. 2, red shaded region). Instead, it shifts into a compositional regime predicted to exist predominantly as a single liquid-ordered (Lo) phase (Fig. 2, red shaded region). Under these conditions, the classical picture of sphingomyelin- and cholesterol-rich domains dispersed within a more fluid membrane does not exist, as the outer leaflet would be expected to be uniformly ordered rather than laterally phase-separated.

In addition, increasing the cholesterol content of exoplasmic leaflet models shifts their composition toward the cholesterol solubility limit in lipid membranes. As an example, the horizontal lines in the phase diagram (Fig. 2 a) represent the maximum solubility limit of cholesterol in the lipid mixtures (blue line: DSPC/DOPC/chol, dark green line: SDPC/chol, and red line: POPE/chol) (Huang et al. 1999). Beyond this limit, cholesterol can no longer fit within the bilayer and instead tends to separate into cholesterol crystals. This constraint should therefore be considered when evaluating models that propose a substantial enrichment of cholesterol in the outer leaflet.

Nevertheless, the implications of these findings depend critically on the magnitude of the proposed imbalance in phospholipids and cholesterol. Although the phospholipid imbalance model offers an alternative perspective on plasma membrane organization, the currently available experimental evidence does not necessarily exclude the existence of sphingomyelin- and cholesterol-rich domains in the exoplasmic leaflet. Indeed, many of the reported observations can also be interpreted within a framework of lateral membrane heterogeneity.

Two important considerations should therefore be kept in mind. First, the experimental observations do not uniquely support a large cholesterol imbalance between the two leaflets; alternative interpretations based on the membrane heterogeneity remain plausible. Second, even if a phospholipid imbalance exists under physiological conditions, its magnitude could be smaller than twofold and therefore insufficient to shift the exoplasmic leaflet from a liquid-ordered/liquid-disordered coexistence regime into a uniformly liquid-ordered phase. Consequently, the available evidence does not yet rule out the possibility that the outer leaflet remains laterally heterogeneous, with sphingomyelin- and cholesterol-rich domains coexisting with more fluid membrane regions.

One of the principal arguments supporting the phospholipid imbalance model concerns the biophysical properties of the cytoplasmic leaflet (Doktorova et al. 2025). The authors propose that this leaflet, being relatively cholesterol-depleted, exhibits reduced lipid packing, elevated membrane tension, and an increased frequency of transient hydrophobic defects—that is, temporary exposures of the membrane's hydrophobic core. Such defects could provide favorable binding sites for lipid-anchored peripheral proteins. To test this hypothesis, the authors monitored the membrane association of the lipidated Lyn SH4 peptide in living cells. Disruption of membrane asymmetry using a calcium ionophore caused the peptide to dissociate from the plasma membrane and redistribute into the cytosol, suggesting that maintenance of leaflet asymmetry is required for stable membrane binding. Similar observations with additional lipidated peptides further support the idea that membrane physical properties influence peripheral protein recruitment.

Although these experiments convincingly demonstrate the importance of membrane asymmetry, they do not uniquely identify phospholipid imbalance as its underlying cause. Hydrophobic defects may arise through multiple mechanisms. For example, nanoscale membrane domains in the exoplasmic leaflet, such as cholesterol- and sphingomyelin-rich lipid rafts, could induce corresponding changes in the opposing cytoplasmic leaflet through interleaflet coupling. Such locally altered membrane environments may likewise generate transient packing defects that stabilize lipidated proteins. Under this interpretation, the subsequent loss of membrane asymmetry induced by lipid scrambling would disrupt these organized domains and their associated packing defects, thereby promoting peptide dissociation. Consequently, the observed experiments demonstrate that membrane organization governs peripheral protein binding but do not distinguish whether this organization originates from a global phospholipid imbalance or from localized membrane domains.

A second line of evidence supporting the phospholipid imbalance model involves manipulation of the outer leaflet phospholipid content (Doktorova et al. 2025). In these experiments, methyl-α-cyclodextrin (MαCD) was used to transfer phospholipids from donor membranes into the exoplasmic leaflet of acceptor cells without inducing detectable membrane scrambling or significant morphological changes. Increasing the phospholipid content of the outer leaflet by approximately 40% resulted in the apparent redistribution of cholesterol toward the cytoplasmic leaflet, as inferred from the recruitment of the cholesterol-sensing protein GRAM-H. Consistent with this observation, the cholesterol transport proteins GRAMD1A and GRAMD1B accumulated at endoplasmic reticulum–plasma membrane contact sites, where they participate in cholesterol transport from the plasma membrane to the endoplasmic reticulum. Furthermore, intracellular lipid-packing sensors detected increased membrane order within internal membranes, consistent with cholesterol redistribution throughout the cell.

These experiments represent an elegant strategy for monitoring cholesterol dynamics in living cells and provide valuable insight into the remarkable moldability of cholesterol distribution. Nevertheless, they do not constitute direct evidence for a large steady-state phospholipid imbalance. Increasing the phospholipid content of the exoplasmic leaflet necessarily perturbs the interactions of cholesterol and the neighboring lipids, irrespective of whether cholesterol is uniformly distributed or partitioned into cholesterol-rich nanodomains. Moreover, artificially increasing the number of phospholipids in only one leaflet introduces a substantial area imbalance that the membrane must compensate for through lipid redistribution, curvature changes, or other relaxation mechanisms. From this perspective, cholesterol redistribution is therefore expected as the membrane reaches a new relaxed state. Consequently, cholesterol movement following phospholipid loading cannot by itself prove the cholesterol imbalance on the exoplasmic leaflet.

A molecular dynamics study of the A2A adenosine receptor further illustrates the importance of membrane asymmetry in modulating cholesterol–protein interactions. In simulations employing an asymmetric plasma membrane model, in which the exoplasmic leaflet was enriched in cholesterol, multiple cholesterol molecules established stable interactions with transmembrane helices TM2, TM6, and TM7. These interactions were proposed to contribute to receptor stabilization within the membrane. In contrast, simulations performed using a symmetric membrane exhibited a markedly different interaction pattern: cholesterol interactions with TM2 and TM7 were substantially reduced, while binding became predominantly localized to TM6 (Ji and Lyman 2025). These differences demonstrate that the native asymmetric lipid composition of the plasma membrane strongly influences the distribution of cholesterol around membrane proteins and, consequently, may affect receptor structure and function.

Interestingly, the simulations did not detect persistent interactions between cholesterol and the proposed cholesterol consensus motif located on the cytoplasmic side of the receptor. Instead, this region interacted preferentially with negatively charged phosphatidylserine lipids, reflecting the characteristic lipid composition of the inner leaflet. Thus, the absence of stable cholesterol contacts on the cytoplasmic face does not necessarily imply that cholesterol is excluded from this leaflet; rather, it highlights the dominant role of phosphatidylserine in mediating protein–lipid interactions at the intracellular membrane interface.

From the perspective of the lipid raft hypothesis, these findings can also be interpreted within the context of lateral membrane heterogeneity. Lipid domains provide localized membrane environments that are substantially enriched in cholesterol, with some studies reporting cholesterol concentrations up to 3- to fourfold higher than those in the surrounding non-domain membrane. Consequently, the high local cholesterol concentrations required to stabilize membrane proteins do not need to arise from a globally cholesterol-rich outer leaflet. However, they may instead be achieved by forming cholesterol-rich domains. Within this framework, the preferential interaction of cytoplasmic-facing residues with phosphatidylserine is fully compatible with domain-mediated organization, as the inner leaflet is expected to maintain its characteristic phosphatidylserine-rich composition while remaining coupled to cholesterol-enriched domains in the opposing leaflet.

If the exoplasmic leaflet is indeed enriched in cholesterol to the extent predicted by phospholipid imbalance ratios exceeding twofold, its composition would be expected to reside predominantly within the liquid-ordered (Lo) region of the phase diagram. Under this scenario, the plasma membrane would consist of a highly ordered, cholesterol-rich outer leaflet coupled to a cytoplasmic leaflet enriched in polyunsaturated phospholipids and characterized by substantially greater fluidity. Such a model therefore implies the coexistence of two monolayers with markedly different physical properties.

Whether such a pronounced asymmetry can be maintained depends critically on the strength of interleaflet coupling. If coupling is weak, each leaflet may largely retain its intrinsic phase behavior. It is also important to note that cholesterol distribution was not measured directly but inferred from phospholipid asymmetry. Consequently, the contribution of membrane proteins to the area balance between the two leaflets cannot be excluded, leaving open the possibility that proteins partially compensate for the inferred phospholipid imbalance and influence estimates of leaflet-specific cholesterol content.

As discussed in later sections, studies using asymmetric model membranes demonstrate that domain formation in one leaflet can induce changes in lipid packing, molecular order, and lateral organization in the opposing leaflet, even when the lipid compositions of the two leaflets differ substantially. These observations indicate that interleaflet interactions tend to reduce, rather than amplify, the physical contrast between the two monolayers. Consequently, models predicting an outer leaflet that is uniformly liquid ordered and an inner leaflet that remains highly fluid implicitly require relatively weak interleaflet interactions. Evaluating the extent of such coupling is therefore essential for determining whether this scenario accurately describes the organization of the plasma membrane.

## Counterpoints of phospholipid imbalance obtained from enzymatic lipid hydrolysis

Recent advances in our understanding of lipid hydrolysis have important implications for the experimental determination of plasma membrane lipid asymmetry. In particular, several studies have highlighted methodological challenges associated with enzymatic digestion assays, which are widely used to estimate the lipid composition of individual membrane leaflets. These findings warrant careful consideration when interpreting reports of a substantial phospholipid imbalance across the plasma membrane. While the phospholipid imbalance model has stimulated important discussion, additional experimental validation using complementary methodologies will be essential to establish both the existence and the magnitude of such an imbalance under physiological conditions.

A recent analysis by (Feigenson 2025) emphasizes that accurate quantification of exoplasmic leaflet lipids requires essentially complete hydrolysis of all accessible phosphatidylcholine (PC) and sphingomyelin (SM) molecules. Achieving this level of digestion, however, is not straightforward because the catalytic activity of membrane-active enzymes depends strongly on the physical state of the membrane. In particular, phospholipase A2 (PLA2) is highly sensitive to monolayer surface pressure. As PC hydrolysis proceeds, the accumulation of reaction products alters the packing properties of the exoplasmic leaflet, increasing monolayer pressure and progressively reducing enzyme activity. Consequently, incomplete hydrolysis may arise not from the absence of substrate but from changes in the physical environment created during the reaction itself. Feigenson suggests that this effect can be minimized by a carefully designed sequential digestion protocol, in which PLA2 first hydrolyzes PC, followed by sphingomyelinase (SMase), whose activity helps relieve the increase in monolayer pressure and promotes more complete lipid digestion. In addition, the activity of lipases is also modulated by the presence of domains (Fanani et al. 2002; De Tullio et al. 2007; Silva et al. 2009).

A second consideration concerns the possibility that the enzymes used to probe membrane asymmetry may themselves perturb the very property they are intended to measure. Studies have shown that sphingomyelinase induces substantial membrane remodeling by generating ceramide, which can alter membrane curvature, lipid packing, and transbilayer lipid movement. In addition, sphingomyelinase treatment has been reported to facilitate the translocation (flip-flop) of several lipid species, including ceramide and gangliosides, across the bilayer (Contreras et al. 2009; Pohl et al. 2009). If similar lipid redistribution occurs during enzymatic digestion assays, lipids originally confined to the exoplasmic leaflet may become redistributed between the two leaflets while the reaction is still in progress. Such membrane remodeling would complicate the interpretation of lipidomic measurements by making the observed lipid distribution reflect both the native membrane organization and enzyme-induced lipid translocation.

To accurately determine transbilayer lipid asymmetry, it is essential to use experimental methods that account for the dynamic interactions among enzyme activity, membrane mechanics, and lipid redistribution. Ongoing methodological improvements will be crucial for quantifying phospholipid asymmetry and assessing its implications for existing models of plasma membrane organization.

## Cholesterol distribution in asymmetric bilayers

The transbilayer distribution of cholesterol remains a subject of considerable debate, both in biological membranes and in asymmetric model systems. Beyond the considerations discussed above, several experimental and theoretical studies have addressed this question, often reaching markedly different conclusions. For example, (Mondal et al. 2009) used leaflet-selective fluorescence quenching of the intrinsically fluorescent sterol analogues dehydroergosterol (DHE) and cholestatrienol (CTL) in CHO cells and concluded that approximately 60–70% of plasma-membrane sterol resides in the cytoplasmic leaflet. However, because native cholesterol was not measured directly, this interpretation relies on the assumption that these fluorescent sterol analogues reproduce the transbilayer distribution and dynamics of cholesterol.

In contrast, (Liu et al. 2017) used engineered PFO-D4-based cholesterol sensors and reported a pronounced asymmetry in the opposite direction, with the apparent cholesterol concentration in the cytoplasmic leaflet approximately 12-fold lower than that in the exoplasmic leaflet. This result, however, also requires careful interpretation because PFO-D4 binding reflects cholesterol accessibility rather than simply the total number of cholesterol molecules present in a leaflet. Sensor binding can be influenced by the local lipid and protein environment, membrane packing, and cholesterol–lipid interactions. Indeed, subsequent work questioned whether the reported difference in sensor response could be directly translated into a 12-fold difference in cholesterol abundance between the two leaflets (Courtney et al. 2018). Thus, these apparently contradictory studies illustrate a central difficulty in resolving cholesterol asymmetry in biological membranes: different experimental approaches may probe different properties of cholesterol, such as its total abundance, accessibility, or chemical environment.

Asymmetric model membranes provide an important complementary platform in which some of these variables can be more carefully controlled. In this context, Zhu et al. (2024b) investigated cholesterol distribution using isotope-labeled lipids in combination with magic-angle spinning (MAS) NMR and small-angle neutron scattering (SANS). Using ^13C-labeled cholesterol, the authors found that membranes containing a saturated high-*T*ₘ lipid (DPPC or SM), POPC, and cholesterol spontaneously developed compositional asymmetry between the two leaflets at equilibrium. Cholesterol preferentially partitioned into the inner leaflet, together with the saturated lipid DPPC or SM, whereas POPC preferentially partitioned into the outer leaflet. In parallel, MAS NMR measurements established that cholesterol undergoes transbilayer flip-flop, although with a slower rate than previously suggested. The agreement between MAS NMR and SANS provided complementary support for the inferred asymmetric distribution. Nevertheless, some limitations should be considered when relating these observations to native cholesterol. Although ^13^C labeling is generally regarded as minimally perturbative, isotope substitution cannot always be assumed to be completely inert; isotope effects, particularly those associated with deuteration, have been shown to modify lipid physical properties, including phase-transition temperatures. In addition, the cholesterol flip-flop rates reported in this study are substantially slower than many values reported elsewhere for cholesterol transbilayer diffusion. Whether these differences arise from membrane composition, experimental methodology, isotope labeling, or a combination of these factors remains to be established.

The problem has also been approached theoretically. Varma and Deserno (2022) combined coarse-grained molecular dynamics simulations with a theoretical framework to investigate cholesterol partitioning between the leaflets of asymmetric lipid bilayers. Their results indicate that cholesterol distribution is determined by an interplay among lipid composition, mixing thermodynamics, and differential lateral stress between the two leaflets, rather than by leaflet area matching alone. In particular, cholesterol can redistribute between leaflets to reduce the free-energy penalty associated with membrane asymmetry, producing a strong relationship between cholesterol partitioning and differential stress. Although this work identifies differential stress as a potentially important driving force for cholesterol redistribution, the predicted strong enrichment of cholesterol in the exoplasmic leaflet remains difficult to establish experimentally, particularly because direct and unambiguous measurements of native cholesterol distribution between the two leaflets of biological membranes remain challenging.

More broadly, theoretical analysis emphasizes an important consideration for studies of asymmetric membranes: differential leaflet stress should be taken into account when constructing and interpreting asymmetric membrane models (Varma and Deserno 2022). This issue is likely to be even more complex in biological membranes. In addition to their highly heterogeneous lipid compositions, plasma membranes contain transmembrane proteins that may occupy different cross-sectional areas in the two leaflets and undergo conformational changes during their functional cycles, potentially altering leaflet-specific packing, lateral pressure, and mechanical stress. Consequently, lipid number or leaflet area matching alone may not be sufficient to define a mechanically balanced biological membrane.

Overall, the contrasting results obtained using sterol analogues, cholesterol-binding sensors, isotope-labeled cholesterol, and theoretical approaches demonstrate that the transbilayer distribution of cholesterol remains an open question rather than a settled property of the plasma membrane. Importantly, recent advances in the preparation and characterization of asymmetric lipid bilayers provide an opportunity to address this problem systematically. Well-controlled model membranes can first be used to disentangle the contributions of lipid composition, cholesterol redistribution, interleaflet coupling, and differential stress, before progressively incorporating proteins and other components characteristic of biological membranes. In this sense, the development of new asymmetric membrane methodologies is expanding what was once a major experimental frontier and may ultimately help bridge the gap between simplified model systems and the complexity of the plasma membrane.

## Interleaflet interactions

It is important to note that “interleaflet coupling” does not yet have a universally accepted definition in the membrane biophysics literature. The term has been used to describe a range of mechanisms through which the two monolayers of a bilayer influence one another, including mechanical, thermodynamic, compositional, and protein-mediated interactions. Despite this diversity of definitions, there is broad experimental evidence that the two leaflets do not behave as completely independent structures but instead communicate through multiple coupling mechanisms.

One well-studied example is membrane pinning, in which the lateral mobility of specific lipids or proteins becomes locally restricted through interactions with intracellular structures, particularly the actin cytoskeleton. Actin filaments can anchor membrane-associated proteins and selected lipids in the cytoplasmic leaflet, thereby creating regions of reduced molecular mobility. This local immobilization perturbs the surrounding membrane, altering lipid packing, diffusion, and membrane order. Importantly, these effects need not remain confined to the inner leaflet. Through the physical continuity of the bilayer, local changes in membrane organization may propagate across the membrane, influencing the lateral organization of the exoplasmic leaflet and potentially stabilizing or modulating cholesterol- and sphingomyelin-rich domains.

Thus, membrane pinning provides a clear example of interleaflet communication, demonstrating that organization within one monolayer can influence the physical behavior of the opposing leaflet (Fujimoto and Parmryd 2017). More broadly, it illustrates the principle that plasma membrane organization emerges not only from the intrinsic properties of each leaflet but also from the dynamic interactions that couple them into a functional membrane.

## Interleaflet interaction in model membranes

Model membranes provide an ideal platform for investigating interleaflet interactions because their lipid composition and experimental conditions can be precisely controlled. In the studies discussed below, asymmetric bilayers were engineered such that one leaflet mimics the exoplasmic monolayer by exhibiting Ld + Lo phase coexistence, whereas the opposing leaflet models the cytoplasmic membrane as a single, fluid phase enriched in unsaturated phospholipids.

These experiments demonstrate that domain formation in one leaflet can induce lateral organization in the opposing leaflet, even when the latter lacks the lipid composition required to form a canonical liquid-ordered phase on its own (Wan et al. 2008; Kiessling et al. 2009; Wang and London 2018; Enoki and Feigenson 2019, 2022). Specifically, liquid-ordered domains in the model exoplasmic leaflet give rise to corresponding “induced ordered domains” in the fluid leaflet. Remarkably, the induced domains align perfectly with those in the opposing leaflet, suggesting that the two monolayers operate in concert rather than independently. This interleaflet coupling demonstrates a significant communication mechanism between them.

In brief, these results were independently obtained by different research groups using distinct experimental approaches to generate asymmetric lipid bilayers. For example, Tamm and co-workers employed a tethered asymmetric supported lipid bilayer system and observed the formation of induced ordered domains in a leaflet composed of brain PC/brain PE/brain PS/cholesterol (1:1:1:0.75), a composition that is expected to remain in a single fluid phase when assembled as a symmetric bilayer. The ordering of this leaflet was induced by coupling to an opposing phase-separated leaflet composed of brain PC/brain SM/cholesterol (2:2:1). Similar observations were reported for asymmetric bilayers consisting of an outer leaflet of egg PC/egg SM/cholesterol (2:2:1), which undergoes phase separation, and an inner leaflet of egg PC/egg PE/egg PS/cholesterol (1:1:1:0.75). Furthermore, in a subsequent study, the authors extended these findings by examining a broader range of lipid compositions, demonstrating that the induction of ordered domains is a robust phenomenon across multiple asymmetric membrane systems (Wan et al. 2008; Kiessling et al. 2009).

In addition, London’s group prepared asymmetric liposomes using methyl-β-cyclodextrin-mediated lipid exchange between vesicles of different lipid compositions. Similar to the studies described above, one leaflet consisted of a lipid mixture that undergoes Ld and Lo phase separation, whereas the opposing leaflet was exchanged to a simplified DOPC/cholesterol mixture that, when present in a symmetric bilayer, is expected to remain in a single fluid phase. The influence of one leaflet upon another was investigated by monitoring the thermal stability of membrane domains using FRET as a function of temperature (Wang and London 2018).

The third set of experiments employed a fundamentally different approach based on the hemifusion of giant unilamellar vesicles (GUVs) with a supported lipid bilayer (SLB) of a distinct lipid composition. Hemifusion is triggered by the addition of calcium ions, resulting in the connection of the outer leaflets of the GUV and the SLB. Lipids are then exchanged between the two membranes by lateral diffusion within the hemifused outer monolayer. The extent of lipid exchange is monitored in real time by fluorescence microscopy using two spectrally distinct lipid analogs, each initially confined to either the GUV or the SLB. For each individual vesicle, the degree of asymmetry is quantified by the extent of dye exchange, thereby determining the percentage of lipid exchange (Enoki and Feigenson 2019; Enoki 2024).

Using this approach, Enoki and Feigenson demonstrated that asymmetric bilayers containing phase-separated mixtures such as DSPC/DOPC/cholesterol, DSPC/DOPC/POPC/cholesterol, or brain SM/DOPC/cholesterol in one leaflet induce the formation of ordered domains in the opposing DOPC/cholesterol leaflet, a composition that is expected to remain homogeneous in a symmetric bilayer. Importantly, because the degree of lipid exchange is quantified for every individual vesicle, these experiments unequivocally demonstrate that the induced domains are not the result of residual high-*Tm* lipids remaining in the exchanged leaflet, but rather arise from interleaflet coupling between leaflets of distinct compositions (Enoki and Feigenson 2019, 2022; Enoki et al. 2021; Feigenson and Enoki 2023).

Moreover, Enoki and Feigenson proposed an additional mechanism underlying the formation of these domains by analyzing membrane lipid packing in asymmetric bilayers using the generalized polarization (GP) of the C-Laurdan probe. Their results indicated that cholesterol redistributes into the region of the DOPC/cholesterol leaflet directly beneath the Lo domain of the opposing leaflet (Enoki and Feigenson 2022; Feigenson et al. 2023). This local enrichment of cholesterol increases lipid packing and membrane order, giving rise to a distinct ordered region despite the absence of high-*Tm* lipids. Based on these findings, they introduced the term “induced ordered domain” to describe this interleaflet coupling-induced ordered phase. This concept emphasizes that the ordered domain is not an intrinsic property of the DOPC/cholesterol leaflet. Instead, it emerges through interleaflet coupling, whereby the presence of an Lo domain in the opposing leaflet promotes a local redistribution of cholesterol molecules, leading to increased lipid packing within the corresponding region of the fluid leaflet.

Interestingly, as discussed below, the mean-field model proposed by (Wagner et al. 2007) also predicts a redistribution of lipids*.* In this framework, the local enrichment of cholesterol associated with the formation of the induced ordered domain helps reduce the energetic penalty arising from the contact between the ordered Lo phase and the disordered DOPC/cholesterol region at the bilayer midplane. Thus, cholesterol redistribution provides a plausible physical mechanism by which interleaflet coupling stabilizes the induced ordered domain.

The physical basis of this coupling is thought to arise from the energetic cost of maintaining mismatched membrane environments across the bilayer. When an ordered region in one leaflet overlies a disordered region in the other, differences in lipid packing, hydrophobic thickness, and membrane elasticity create an unfavorable energetic penalty at the bilayer midplane. The membrane lowers this free-energy cost by redistributing lipids laterally within a leaflet, thereby generating a domain of increased molecular order directly beneath the pre-existing liquid-ordered domain. Consequently, even a leaflet that would otherwise exist as a homogeneous fluid can acquire lateral heterogeneity through coupling to an ordered opposing leaflet.

These findings provide compelling experimental evidence that membrane domains can span an asymmetric bilayer despite the two leaflets having markedly different lipid compositions. More broadly, they demonstrate that the organization of the plasma membrane cannot be understood by considering each leaflet in isolation; rather, it emerges from the dynamic interplay between the intrinsic properties of each monolayer and the physical interactions that couple them across the bilayer.

**Fig. 3:**
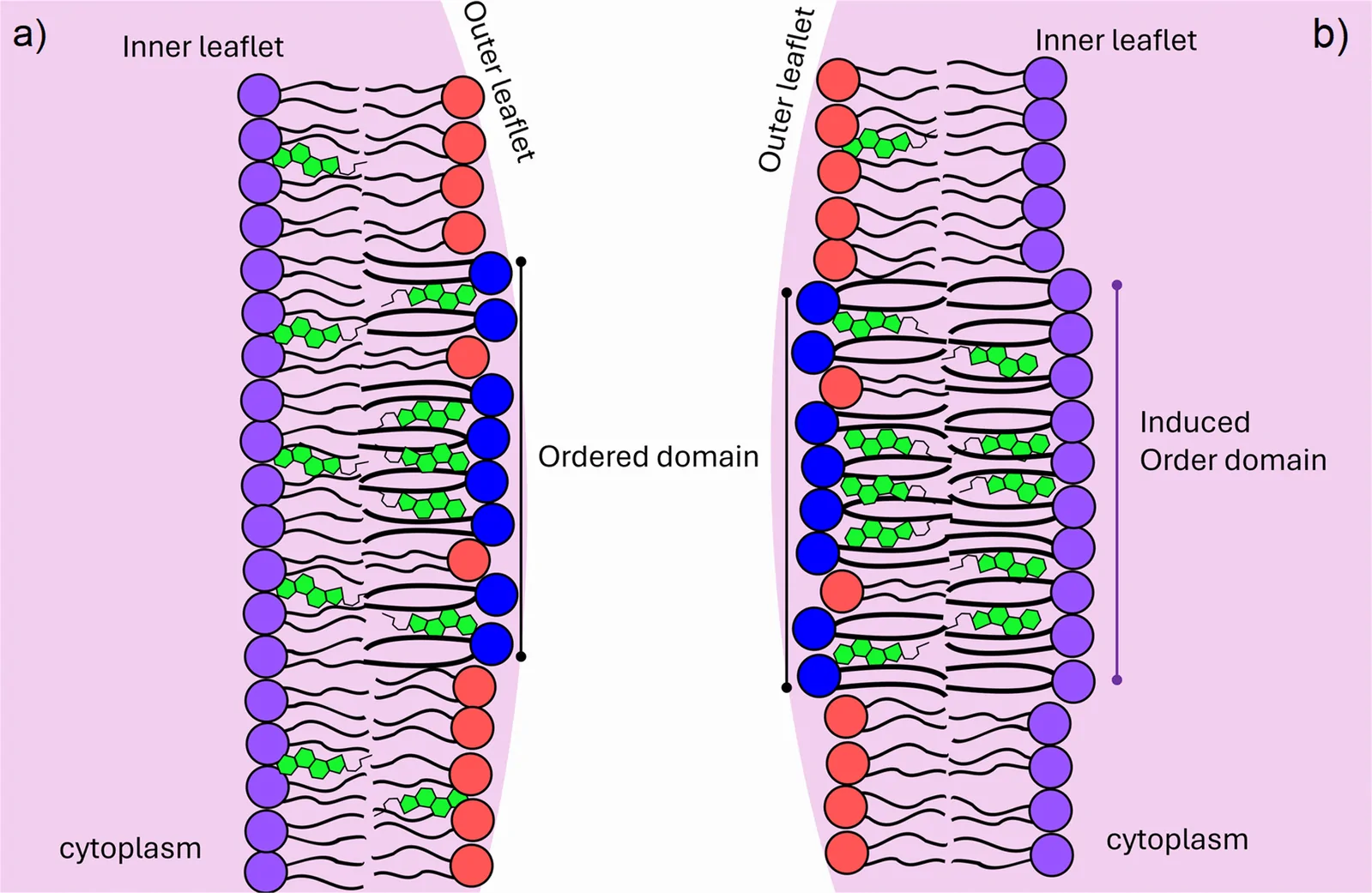
Interleaflet interactions yield induced domains. Illustration of asymmetric bilayers, where the outer leaflet forms domains (Ld + Lo phases), whereas the inner leaflet is highly fluid. (**a**) The representation shows leaflets with minimal interactions, preserving their own properties. (**b**) The sketch illustrates the ordered phase, Lo, on the outer leaflet, inducing an ordered domain in a leaflet that would not form such a domain in the absence of a coupling interaction with the upper leaflet

Figure 3 illustrates two contrasting scenarios for membrane organization in an asymmetric bilayer. In Fig. 3a), the exoplasmic model leaflet contains coexisting liquid-ordered (Lo) and liquid-disordered (Ld) phases, whereas the cytoplasmic model leaflet remains uniformly fluid. Such an arrangement assumes minimal interleaflet coupling, allowing each leaflet to organize independently. Under this scenario, the presence of an ordered domain in one leaflet has little or no influence on the organization of the opposing leaflet. Although this representation is conceptually useful, direct experimental evidence supporting completely independent leaflets has not yet been found. In a similar scenario, the outer leaflet could form a single ordered phase and the inner leaflet a single disordered phase, with negligible inter-leaflet interaction.

In contrast, Fig. 3b) summarizes the behavior observed in asymmetric model membranes. Here, a liquid-ordered domain in the exoplasmic model leaflet induces the formation of a corresponding ordered domain in the otherwise fluid leaflet through interleaflet coupling. This induced domain becomes enriched in cholesterol despite the absence of high-Tm lipids. These findings suggest that lipid rafts in the outer leaflet cell membranes could organize the opposing leaflet by promoting the local redistribution of cholesterol and aminophospholipids. Consequently, phosphatidylethanolamine (PE) and phosphatidylserine (PS) may partition into distinct membrane environments, giving rise to transbilayer domains that are compositionally asymmetric yet physically coupled.

Unlike biological membranes, which actively maintain lipid asymmetry through energy-dependent processes, our asymmetric model bilayers are passive systems that ultimately evolve toward thermodynamic equilibrium. As a consequence, lipid asymmetry is expected to dissipate over time as lipids undergo transbilayer flip-flop, eventually leading to a symmetric bilayer. However, numerous experimental studies have demonstrated that spontaneous phospholipid flip-flop in model membranes is an intrinsically slow process, with characteristic half-times ranging from several days to weeks, depending on lipid composition and experimental conditions. Therefore, the timescale required for complete relaxation of membrane asymmetry is orders of magnitude longer than that of our experiments, which are completed within a few hours. Under these conditions, phospholipid redistribution across the bilayer is negligible, and the membrane can be regarded as maintaining a stable asymmetric composition. Accordingly, although the system is not in true thermodynamic equilibrium, it remains in a well-defined quasi-equilibrium state throughout the experiments, providing a reliable framework for investigating the effects of membrane asymmetry.

In contrast, related experiments using asymmetric model membranes have shown that modifying the properties of the ordered leaflet can produce the opposite effect (Wang and London 2018; London 2019; Enoki and Heberle 2023). In our studies employing the hemifusion method, the acyl-chain length of the high-melting-temperature (high-Tm) lipid in the phase-separating leaflet was systematically varied (18:0-PC, 17:0-PC, 16:0-PC, and 15:0-PC), while the opposing leaflet was kept constant and composed of unsaturated phosphatidylcholine and cholesterol (data under review). As the chain length of the high-Tm lipid decreased, the influence of the fluid leaflet became increasingly pronounced. Rather than inducing order in the opposing leaflet, the fluid leaflet progressively suppressed phase separation in the opposing leaflet, ultimately favoring a more homogeneous membrane.

These observations demonstrate that interleaflet coupling is bidirectional: one leaflet can either promote or suppress lateral organization in the other, depending on the balance of molecular interactions across the bilayer. To provide a theoretical framework for interleaflet coupling, (Wagner et al. 2007) developed a minimal mean-field model describing the lateral phase behavior of asymmetric lipid bilayers. In this model for a binary, each leaflet is represented by a single compositional parameter, denoted by ϕ and ψ for the upper and lower leaflets, respectively. The free energy of the bilayer is expressed as

1 $$f\left(\phi,\psi\right)=f_{BW}\left(\phi\right)+f_{BW}\left(\psi\right)+\Lambda{(\phi-\psi)}^2$$

where ${f}_{BW}\left(\phi \right)$ and ${f}_{BW}\left(\psi \right)$ are the Bragg–Williams (regular solution, ${f}_{BW}\left(\phi \right)=\phi ln\phi +\left(1-\phi \right)\mathrm{ln}\left(1-\phi \right)+\chi \phi (1-\phi )$) free energy of an individual two-component monolayer, where χ is the effective strength of the lateral neighbor interaction. The final term of Eq. (1) introduces an energetic penalty for local compositional differences between opposing leaflets. The coupling parameter, Λ, therefore quantifies the strength of interleaflet interactions, favoring similar local compositions across the bilayer.

This simple framework predicts distinct outcomes depending on the thermodynamic stability of the two leaflets. A phase-separated leaflet can induce phase separation in an opposing leaflet that would otherwise remain homogeneous, whereas a sufficiently stable homogeneous leaflet can suppress phase separation in an intrinsically phase-separating leaflet. In addition, the model predicts that interleaflet coupling promotes domain registration and, under certain coupling conditions, can give rise to more complex morphologies involving partial domain registration and three-phase coexistence. In this case, the three coexisting bilayer phases correspond to a registered ordered/ordered phase, a registered disordered/disordered phase, and an unregistered ordered/disordered phase.

Interestingly, the model further predicts that lipid compositions redistribute laterally to minimize the free-energy penalty associated with compositional mismatch across the bilayer. This prediction is remarkably consistent with the experimental observations of Enoki and Feigenson, who demonstrated that cholesterol redistributes into the fluid DOPC/cholesterol leaflet during the formation of the induced ordered domain. This local enrichment of cholesterol increases lipid packing within the corresponding region of the fluid leaflet while reducing the energetic penalty associated with the contact between an ordered Lo domain and a disordered Ld region across the bilayer midplane. Together, these findings provide a plausible physical mechanism for the formation and stabilization of induced ordered domains.

Importantly, interleaflet coupling does not necessarily require direct physical contact between hydrocarbon chains, such as chain interdigitation. Nevertheless, when such contact occurs, it may strengthen the coupling between opposing leaflets and further promote their cooperative lateral organization (Frewein et al. 2022).

Finally, the limiting case of vanishing interleaflet coupling (Λ → 0) implies that the two leaflets behave as independent thermodynamic systems, each undergoing lateral organization according to its own composition, with no influence from the opposing leaflet. To the best of our knowledge, however, experimental studies of asymmetric lipid bilayers have not provided evidence supporting completely independent leaflet behavior.

Although the quasi-equilibrium state reached in our biomimetic membrane systems may favor cholesterol redistribution or the formation of induced ordered domains, the pathways by which membranes reach these states may involve significant kinetic barriers. Recent theoretical work has proposed that lipid transport between membranes may proceed through rare cooperative rearrangements rather than through independent lipid motions, (Wang et al. 2026). This perspective suggests that membrane organization is governed not only by thermodynamic driving forces but also by the kinetics of lipid transport, cholesterol redistribution, and the relaxation of membrane asymmetry. Integrating equilibrium and kinetic descriptions may therefore provide a more complete understanding of membrane organization and interleaflet coupling in biological membranes.

Together, these results highlight that interleaflet coupling is highly sensitive to membrane composition, particularly to the properties of the high-Tm lipids that stabilize the ordered phase. More importantly, they demonstrate that the organization of an asymmetric bilayer cannot be inferred from the behavior of its individual leaflets in isolation. Instead, membrane structure emerges from the competition between intra- and interleaflet interactions, with either leaflet capable of dominating the phase behavior of the bilayer under different compositional conditions (Enoki and Heberle 2023).

Additional insight into the role of membrane asymmetry has come from studies of giant plasma membrane vesicles (GPMVs). New reports have shown that the appearance of micron-sized membrane domains accompanies the induced loss of lipid asymmetry in GPMV (Kakuda et al. 2022). These observations have prompted the intriguing question of whether the plasma membrane already contains nanoscale domains before asymmetry is lost, with membrane asymmetry acting either to limit their sizes or to prevent their formation altogether. At the same time, it is important to recognize that the isolation procedures used to generate GPMVs alter the native state of the plasma membrane. Consequently, the lipid composition and transbilayer organization of GPMVs may not fully reflect those of intact cells, and caution is therefore warranted when extrapolating these observations to the native plasma membrane.

The loss of asymmetry itself is expected to produce profound changes in membrane organization. Redistribution of sphingomyelin and other high-Tm lipids between the two leaflets enables both monolayers to adopt lipid compositions capable of Ld + Lo phases. Under these conditions, domains formed in each leaflet can become laterally registered, producing transbilayer domains that are larger and more stable than those expected in the native asymmetric membrane.

From a biophysical perspective, this behavior is also consistent with changes in line tension. The boundary between coexisting Ld and Lo phases carries an energetic cost that depends, in part, on differences in lipid packing and hydrophobic thickness across the phase interface. When both leaflets undergo phase separation, the registration of ordered domains across the bilayer can increase the energetic driving force for domain coalescence, thereby favoring the formation of larger, micron-sized domains. In contrast, an asymmetric membrane containing a liquid-ordered domain in only one leaflet and an induced domain in the opposing leaflet is expected to exhibit a lower effective line tension because the opposing leaflet does not contain an intrinsic Ld/Lo interface. Reduced line tension favors smaller domain sizes, providing a plausible physical explanation for why asymmetric membranes may contain nanoscopic domains, whereas loss of asymmetry promotes the emergence of optically resolvable micron-sized domains. Thus, the observation of large domains following the loss of membrane asymmetry does not necessarily argue against the existence of nanodomains in intact plasma membranes.

Finally, we briefly examine an important constraint that must be considered when evaluating the phospholipid (and cholesterol) imbalance model. Specifically, if the exoplasmic leaflet contains high cholesterol fraction, the membrane composition may approach the cholesterol solubility limit, beyond which excess cholesterol separates from the bilayer to form cholesterol crystals. This constraint provides an independent framework for assessing the plausibility of the proposed cholesterol enrichment in the outer leaflet.

## Protein–lipid interactions in membrane organization

As briefly mentioned above, the presence of membrane proteins introduces an additional level of complexity that requires careful consideration, even in in vitro model systems. In addition to lipid–lipid interactions, membrane organization may be influenced by leaflet-specific lipid–protein interactions, lateral pressure profiles induced or sensed by membrane proteins, hydrophobic matching, and mechanical stresses. In this review, we have deliberately focused on developing an understanding of lipid–lipid interactions, with lipid asymmetry as a central element. Building upon this framework, a subsequent challenge will be to incorporate specific lipid–protein interactions and determine how they modify, reinforce, or compete with lipid-driven membrane organization. Although a comprehensive discussion of lipid–protein interactions is beyond the scope of this review, we have included a brief description of two relevant conceptual frameworks: one primarily centered on collective lipid organization, represented here by the lipid-raft hypothesis, and another centered on the local lipid environment of membrane proteins, represented by the concept of annular lipids and, more broadly, preferential lipid–protein interactions (Lee 2003; Dutta et al. 2026).

The lipid-raft framework has been discussed extensively throughout this review. In contrast, the annular-lipid concept proposes that membrane proteins can be surrounded by a lipid environment that differs from the bulk membrane, as preferential protein–lipid interactions generate a local shell enriched in particular lipid species and characterized by distinct packing and dynamics (Lee 2003). More recently, this picture has been broadened by considering the membrane as the solvent environment of membrane proteins, such that preferential solvation by particular lipid species provides a thermodynamic basis for local lipid enrichment around proteins (Dutta et al. 2026). Although both concepts can therefore lead to the apparently similar picture of a membrane protein surrounded by a compositionally or physically distinct lipid environment, the physical principles underlying this organization are fundamentally different. Annular lipids generally constitute a dynamic protein-associated shell in which individual lipids continuously exchange with the surrounding membrane, with local enrichment arising primarily from preferential lipid–protein interactions. Lipid rafts, in contrast, arise from cooperative lipid–lipid and lipid–protein interactions and can, under appropriate conditions, reflect thermodynamically favored lateral membrane organization. An annular lipid shell should therefore not be equated with a lipid raft.

An important question, therefore, is how these two conceptually distinct mechanisms can be reconciled within a broader picture of membrane organization. Preferential interactions of a protein with cholesterol, sphingolipids, or other lipids associated with ordered environments could locally enrich these components and potentially contribute to the nucleation or stabilization of raft-like organization. Conversely, a thermodynamically favored ordered lipid environment may promote the partitioning of proteins whose lipid-interaction preferences are compatible with that environment. Thus, while describing annular lipids and lipid rafts within a single unified framework may not be straightforward, considering both concepts emphasizes the importance of developing a more complete picture in which lipid–lipid interactions, lipid asymmetry, and specific lipid–protein interactions collectively determine membrane organization.

## Summary and perspectives

The study of plasma membrane asymmetry is entering an exciting phase, driven by advances in lipidomics, molecular simulations, and the development of increasingly sophisticated model membrane systems. These complementary approaches are refining our understanding of membrane organization, yet many fundamental questions remain unresolved. Rather than converging on a single definitive model, recent discoveries have revealed that the plasma membrane is more complex than previously appreciated. Each new experimental and theoretical result contributes an important piece to a larger puzzle whose complete picture has yet to emerge.

Over the past three decades, the lipid raft hypothesis has provided one of the most influential frameworks for interpreting plasma membrane organization. Its strength lies not only in its biophysical foundation, established through studies of model membranes, but also in its ability to explain a wide range of biological observations, including membrane trafficking, protein partitioning, signal transduction, and host–pathogen interactions. At the same time, recent studies proposing a phospholipid (and cholesterol) imbalance between the two leaflets have introduced an alternative perspective.

The increasing evidence derived from studies on asymmetric model membranes indicates that lipid rafts can be reconciled with bilayer asymmetry through the mechanism of interleaflet coupling. In this framework, Lo domains in the exoplasmic leaflet can induce the formation of ordered domains in the distinct cytoplasmic leaflet. This interaction enables a cohesive transbilayer membrane organization to develop, despite the significantly different lipid compositions of the two monolayers. Such coupling provides a plausible physical mechanism by which lateral membrane heterogeneity can coexist with the intrinsic asymmetry of the plasma membrane.

More broadly, our discussion highlights the central role of interleaflet interactions in determining membrane organization. These interactions have received considerably less attention than lateral lipid segregation within individual leaflets. However, a growing number of experimental and theoretical studies now indicate that the two monolayers can communicate, such that the organization of one leaflet can influence the other. Although the underlying mechanisms and their physiological consequences remain incompletely understood, these findings suggest that interleaflet coupling should be considered a fundamental component of any comprehensive model of plasma membrane structure.

The recently proposed phospholipid imbalance model has stimulated important discussion within the membrane biophysics community. Nevertheless, further experimental work will be required to establish the magnitude of any phospholipid imbalance under physiological conditions and to determine whether it is sufficient to alter the phase behavior of the exoplasmic leaflet. Resolving this question will require complementary experimental approaches capable of accurately quantifying leaflet-specific lipid composition while minimizing perturbations to membrane asymmetry.

Finally, one fundamental question remains at the core of plasma membrane organization. The exoplasmic leaflet is uniquely enriched in sphingomyelin to an extent unmatched by any other cellular membrane. What evolutionary advantage does this remarkable enrichment confer? Is sphingomyelin primarily required to generate cholesterol-rich membrane domains that organize the plasma membrane, or does it instead serve to solubilize and stabilize the high cholesterol content of the outer leaflet? Answering this question will not only clarify the role of sphingomyelin but may also provide the key to understanding how the plasma membrane achieves its unique structural and functional properties.

## Supplementary Information

Below is the link to the electronic supplementary material.

ESM 1 (PDF 344 KB)

## Data Availability

No datasets were generated or analyzed during the current study.
