# Supplementary material for: Domains or no domains? That is the question: insights from asymmetric bilayer models into plasma membrane lipid organization
Source: Biophys Rev. 2026 Sep 30;18(5):927–46. doi: 10.1007/s12551-026-01468-1 (PMC13751941; doi:10.1007/s12551-026-01468-1)

“Domains or No Domains? That Is the Question: Insights from Asymmetric Bilayer Models into Plasma Membrane Lipid Organization”

Thais Azevedo Enoki (corresponding author: enokita@if.usp.br)

Universidade de São Paulo, Institute of Physics, Department of Experimental Physics, São Paulo, SP, Brazil05508-090.

Supporting Material

Phospholipid composition varies substantially among cellular organelles, reflecting their distinct structural and functional requirements. Phosphatidylcholine (PC) is abundant throughout cellular membranes, whereas phosphatidylethanolamine (PE) is also broadly distributed but particularly enriched in mitochondria and the endoplasmic reticulum. Specific organelles contain characteristic lipids: cardiolipin (CL) is a hallmark of mitochondrial membranes, bis(monoacylglycero)phosphate (BMP) is enriched in late endosomes, and sphingomyelin (SM) is particularly enriched at the plasma membrane. Overall, these compositional differences contribute to organelle-specific membrane properties, including curvature, packing, fluidity, and interactions with membrane-associated proteins.

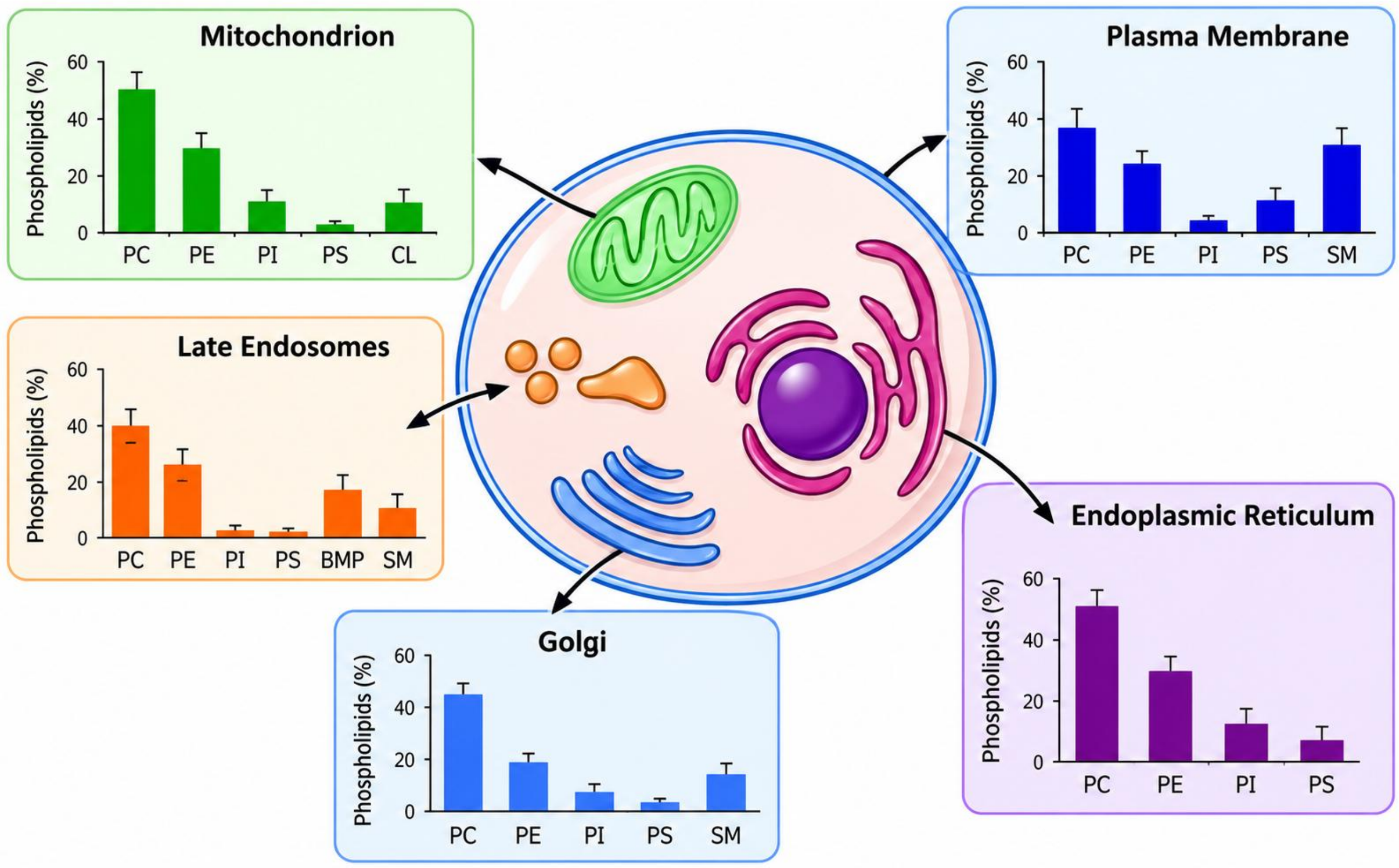

Figure S1. Distinct lipid composition of cell organelles. The sketch represents a mammalian cell and organelles. The histograms represent the fraction of lipids in the plasma membrane (blue),

mitochondria (green), late endosomes (orange), Golgi apparatus (light blue), and Endoplasmic Reticulum (purple). The fraction of other minor components is not depicted. These fractions are sourced from (van Meer et al. 2008).

Graphical Abstract

The graphical abstract draws on William Shakespeare's famous phrase, **"To be, or not to be, that is the question."** In this review, we explore this idea in the context of plasma membrane organization by examining different frameworks in which lipid domains may—or may not—be an integral feature of membrane structure.

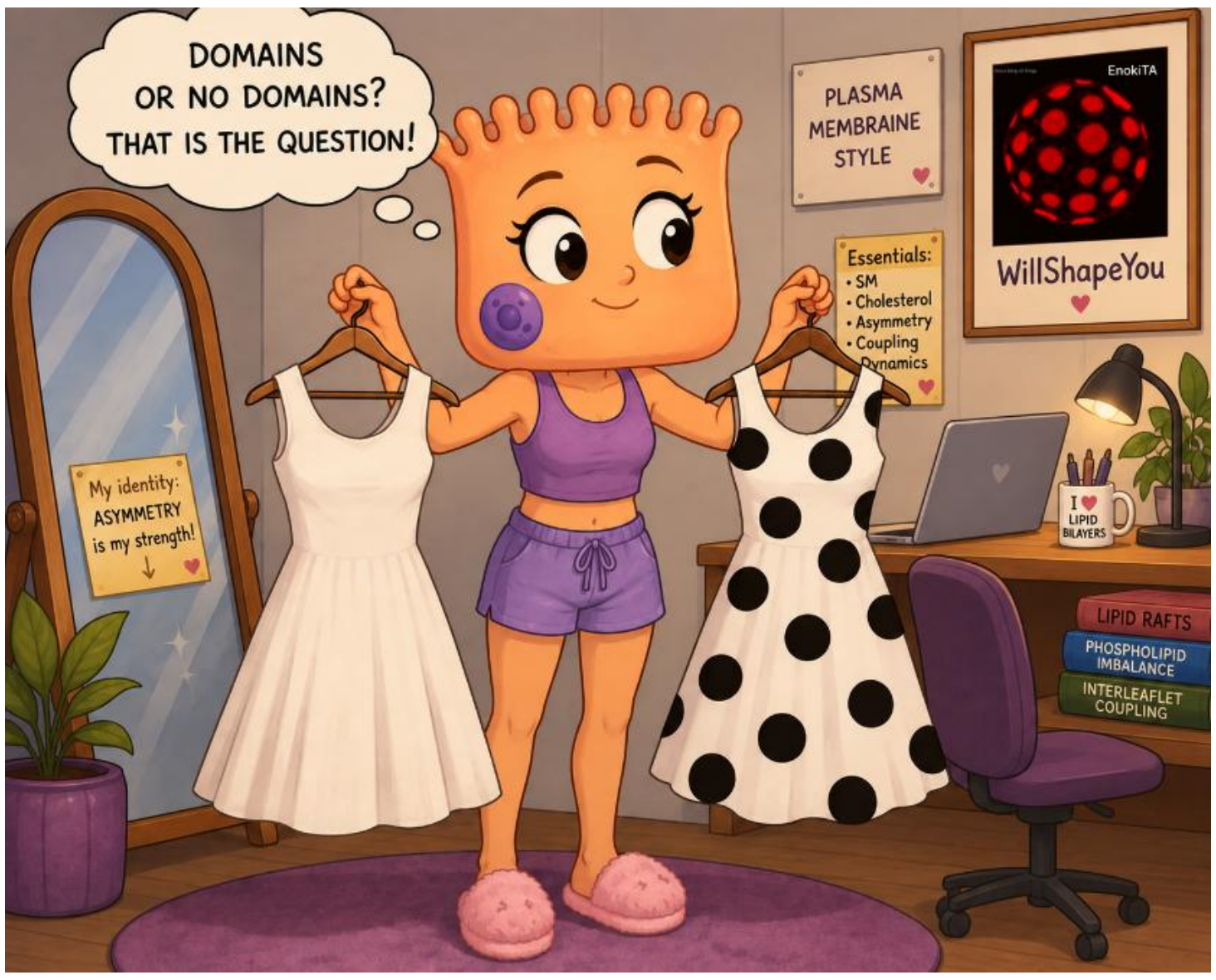

Supplement: Supplementary file 1 [file 12551_2026_1468_MOESM1_ESM.pdf]
